# Morular Metaplasia in Fundic Gland Polyps—A Case Report of a Rare Finding in a Common Lesion

**DOI:** 10.1155/crip/9126498

**Published:** 2026-04-25

**Authors:** Benjamin Kennedy, Deepti Jacob, Kerry Bernal, Geoffrey Talmon

**Affiliations:** ^1^ University of Nebraska Medical Center College of Medicine, Nebraska Medical Center, Omaha, Nebraska, USA; ^2^ Midwest Gastrointestinal Associates, Omaha, Nebraska, USA; ^3^ University of Nebraska Medical Center Department of Pathology, Microbiology, and Immunology, Nebraska Medical Center, Omaha, Nebraska, USA

**Keywords:** case report, fundic gland polyps, morular metaplasia, stomach, Wnt/beta-catenin

## Abstract

**Background:**

Morular metaplasia is a phenomenon described in neoplasms of various sites, including endometrioid neoplasms of the uterus and colonic tubular adenomas. Although of questionable biological significance, they may be confused with squamous differentiation/neoplasia or neuroendocrine lesions.

**Case Presentation:**

A 34 year‐old female patient on proton‐pump inhibitor therapy underwent esophagogastroduodenoscopy for evaluation of bloating and reflux‐type symptoms, which revealed three mucosal polyps within the proximal stomach. Microscopic examination showed conventional fundic gland polyps with foci of squamoid nests of whorled cells. These cells were positive for CDX2, demonstrated abnormal beta‐catenin staining, and were negative for neuroendocrine markers. The diagnosis is fundic gland polyps with morular metaplasia.

**Conclusion:**

This case expands the types of lesions in which morular metaplasia may be identified, particularly in the setting of lesions with abnormalities of the Wnt/beta‐catenin pathway protein expression, and raises awareness of the finding that may be confused with neoplastic lesions.

## 1. Introduction

Fundic gland polyps (FGPs) are among the most common benign lesions occurring in the stomach, seen in up to 2% of patients undergoing esophagogastroduodenoscopy (EGD) [1] and accounting for approximately 80% of gastric polyps [2]. They typically arise in two settings, in association with polyposis syndromes such as familial adenomatous polyposis (FAP) or MUTYH‐associated polyposis or sporadically, the latter often linked to proton‐pump inhibitor therapy [2].

The development of squamous‐like morules has been described in numerous other lesions at various sites, including the endometrium, colon, and thyroid. Thought to represent a metaplastic phenomenon by some authors with questionable therapeutic significance [3], their presence in a FGP is extremely rare and may cause diagnostic confusion with other entities arising in the stomach such as neuroendocrine or squamous neoplasms. Herein, we report a case of a FGP containing morules. The immunophenotype of this finding is also presented.

## 2. Case Presentation

A 34‐year‐old female with a history of irritable bowel syndrome (IBS), ulcer disease, and a family history of IBS presented with complaints of episodic stomach bloat and a burning sensation in her chest. She was taking pantoprazole at presentation. Family history was noncontributory.

She underwent an EGD that revealed normal mucosa in the esophagus and the duodenum, with a few small sessile polyps in the stomach fundus and body, which were biopsied. Pathologic examination revealed focal surface atypia and acute inflammation and no other microscopic findings. Since low‐grade epithelial dysplasia could not be ruled out, complete polypectomy was recommended.

**Figure 1:**
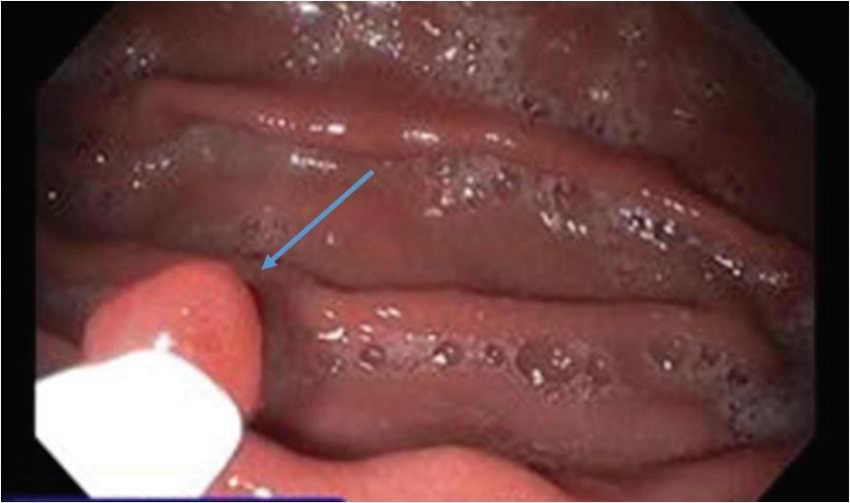
One of the three mucosal‐based polyps within the body of the stomach (arrow) measuring 0.3 cm in greatest dimension.

**Figure 2:**
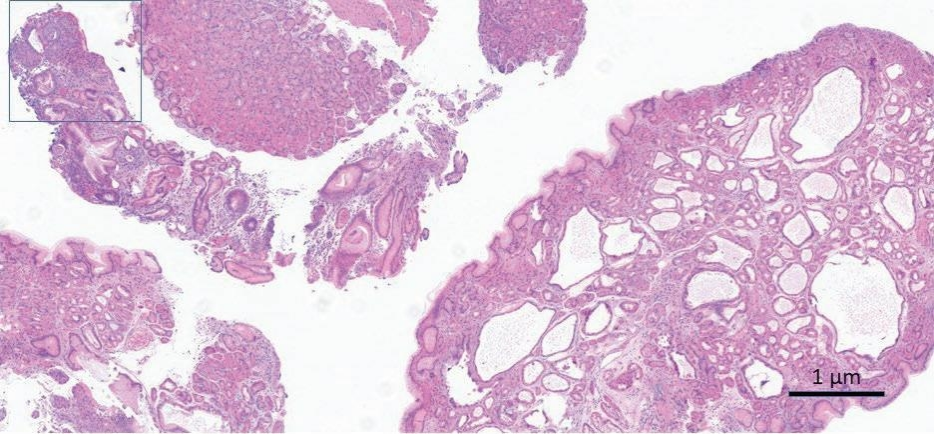
Low power image of fundic gland polyps showing dilated oxyntic glands and stromal edema. The focus demonstrating morular metaplasia was associated with erosion (box: H&E, 25×).

**Figure 3:**
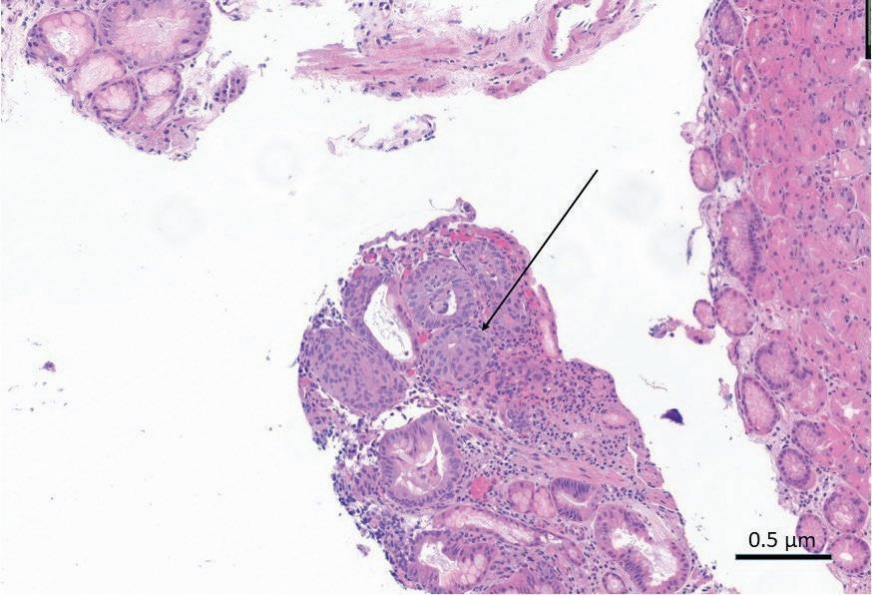
Fragment of fundic gland polyp with rounded cluster of epithelioid cells (arrow) with squamoid morphology, negative for cytologic atypia and mitotic activity (H&E, 100×).

**Figure 4:**
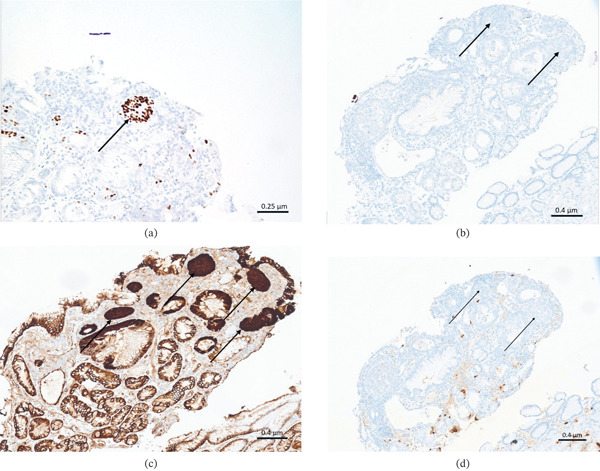
The epithelioid cells (arrows) were positive for CDX2 (a), negative for p40 (b), demonstrated strong cytoplasmic staining for *β* ‐catenin (c), and did not express chromogranin A (d) (all images at 100×).

The patient returned for a second EGD about 2 months later, which revealed three polyps in the proximal stomach ranging from 0.3 to 0.9 cm; these were removed by polypectomy (Figure 1). The remainder of the endoscopic examination was unremarkable. Microscopic examination revealed polypoid fragments of fundic‐type mucosa with dilated oxyntic glands and associated stromal edema (Figure 2). Rare, rounded nests of bland squamoid morules with a whorled growth pattern were seen associated near the superficial epithelial surface and adjacent to areas of erosion involving < 5% of the examined sections of the polyp (Figure 3). Keratinization, necrosis, and mitoses were absent within the nests, and there was no evidence of significant surface epithelial atypia or dysplasia within any tissue fragment. The morular cells were positive for CDX2, CD10 (membranous), and beta‐catenin (cytoplasmic), whereas negative for CK5/6, chromogranin, synaptophysin, p40, and Ki67 (Figure 4a–d). The final diagnosis was FGPs with morular metaplasia. The patient did not return for follow‐up.

## 3. Discussion

FGPs are common benign lesions in the fundus and superior body of the stomach, felt to represent a benign hyperplastic proliferation of oxyntic glands. They arise in two settings, sporadic and syndromic. Sporadic FGPs are more common in females in the fifth and sixth decade of life with a strong positive correlation with proton‐pump inhibitor therapy [1]. FGPs are also associated with syndromes such as FAP; gastric adenocarcinoma and proximal polyposis of the stomach (GAPPS); and MUTYH‐associated polyposis [4–7]. These lesions occur earlier in life, do not demonstrate gender predilection, are often multiple, and are more likely to harbor dysplasia [2].

FGPs arise from the oxyntic mucosa surface and are usually small with a mean diameter of 5–6 mm, demonstrating a smooth surface [2]. Microscopically, the lesions are composed of cystically dilated glands lined by flattened parietal cells, chief cells, and mucous neck cells. When not involved by epithelial dysplasia, the overlying surface epithelium is intact and demonstrates no significant architectural distortion.

Genetic abnormalities are more common in syndromic FGPs, with mutations in *APC* seen in approximately 50% of lesions compared with < 10% of sporadic polyps [8]. Conversely, sporadic lesions are more likely to demonstrate alterations in the CTNNB1 (*β*‐catenin) gene [9]. Both genes are involved in the Wnt/*β*‐catenin signaling pathway, which likely plays a role in the development of many FGPs [6].

Morules are small clusters of uniform epithelioid cells that do not demonstrate significant cytologic atypia or mitotic activity. While appearing squamous‐like, these morules do not stain positively for markers of squamous differentiation such as p40 and p63 [3, 10, 11] or demonstrate intercellular bridges or keratin formation. Although they are somewhat morphologically similar to neuroendocrine cell proliferations, they are typically negative for markers of this differentiation such as chromogranin or synaptophysin.

Morule formation has been associated with tumors demonstrating abnormalities in the Wnt/*β*‐catenin pathway, being described in a subset of endometrioid neoplasms of the gynecologic tract, tubular adenomas, and cribriform morular thyroid carcinoma [10, 12–14] and may serve as a morphologic clue that a lesion possesses an alteration in the signaling pathway. This hypothesis is supported by the abnormal *β*‐catenin staining pattern that has been noted within morular cells [11].

Morules in FGPs are poorly described, with just two prior reports to date. Both were identified in adults with symptoms of gastric reflux [11, 14] and one in the setting of proton‐pump inhibitor therapy. Neither case was associated with a polyposis syndrome. The morules seen in one patient showed nuclear positivity for *β*‐catenin and CDX2, membranous positivity for CD10, and were negative for Ki‐67 as in this case [3]. The other demonstrated focal expression of cytokeratin 5, cytokeratin 6, and synaptophysin, with a similarly low Ki‐67 proliferative index [11]. No report disclosed the presence of associated dysplasia or invasive carcinoma.

Based on the finding′s rarity, the clinical significance of the finding of morules in FGPs is uncertain. In both prior cases, the lack of associated dysplasia or carcinoma supports the contention that they represent an incidental finding related to the underlying genetic abnormality driving the lesion′s development rather than one of specific diagnostic importance. However, experience is limited due to the small number of reported cases.

Herein, we present the case of a rare finding noted in a gastric lesion commonly encountered in surgical pathology practice. Its recognition by pathologists is important to not confuse it with other processes, such as reactive squamous metaplasia [15] or true squamous cell carcinoma [16]. Further, morular metaplasia may serve as a morphologic marker of the lesion′s genetic makeup.

## Author Contributions

B.K.: manuscript drafting and literature review. D.J.: data collection. K.B.: data collection. G.T.: manuscript drafting and data collection.

## Funding

No funding was received for this manuscript.

## Ethics Statement

This case report has been completely anonymized, and all tissue was obtained as part of the standard of care for the patient. The study was not deemed to require patient consent or qualify as human subject research by the University of Nebraska Medical Center Institutional Review Board.

## Conflicts of Interest

The authors declare no conflicts of interest.

## Data Availability

The data that support the findings of this study are available from the corresponding author upon reasonable request.

## References

[1] Sano W. , Inoue F. , Hirata D. , Iwatate M. , Hattori S. , Fujita M. , and Sano Y. , Sporadic Fundic Gland Polyps With Dysplasia or Carcinoma: Clinical and Endoscopic Characteristics, World Journal of Gastrointestinal Oncology. (2021) 13, no. 7, 662–672, 10.4251/wjgo.v13.i7.662, 34322195. 34322195 PMC8299935

[2] Costa D. , Ramai D. , and Tringali A. , Novel Classification of Gastric Polyps: The Good, the Bad and the Ugly, World Journal of Gastroenterology. (2024) 30, no. 31, 3640–3653, 10.3748/wjg.v30.i31.3640, 39192997. 39192997 PMC11346164

[3] Petris G. D. and Chen L. , Morules in Fundic Gland Polyposis: A Case Report, International Journal of Clinical And Experimental Pathology,. (2014) 7, no. 3, 1241–1245, PMID: 24695818; PMCID: PMC3971335. 24695818 PMC3971335

[4] Burt R. W. , Gastric Fundic Gland Polyps, Gastroenterology. (2003) 125, no. 5, 1462–1469, 10.1016/j.gastro.2003.07.017, 2-s2.0-0242406863. 14598262

[5] Li J. , Woods S. L. , Healey S. , Beesley J. , Chen X. , Lee J. S. , Sivakumaran H. , Wayte N. , Nones K. , Waterfall J. J. , Pearson J. , Patch A. M. , Senz J. , Ferreira M. A. , Kaurah P. , Mackenzie R. , Heravi-Moussavi A. , Hansford S. , Lannagan T. R. M. , Spurdle A. B. , Simpson P. T. , da Silva L. , Lakhani S. R. , Clouston A. D. , Bettington M. , Grimpen F. , Busuttil R. A. , di Costanzo N. , Boussioutas A. , Jeanjean M. , Chong G. , Fabre A. , Olschwang S. , Faulkner G. J. , Bellos E. , Coin L. , Rioux K. , Bathe O. F. , Wen X. , Martin H. C. , Neklason D. W. , Davis S. R. , Walker R. L. , Calzone K. A. , Avital I. , Heller T. , Koh C. , Pineda M. , Rudloff U. , Quezado M. , Pichurin P. N. , Hulick P. J. , Weissman S. M. , Newlin A. , Rubinstein W. S. , Sampson J. E. , Hamman K. , Goldgar D. , Poplawski N. , Phillips K. , Schofield L. , Armstrong J. , Kiraly-Borri C. , Suthers G. K. , Huntsman D. G. , Foulkes W. D. , Carneiro F. , Lindor N. M. , Edwards S. L. , French J. D. , Waddell N. , Meltzer P. S. , Worthley D. L. , Schrader K. A. , and Chenevix-Trench G. , Point Mutations in Exon 1B of APC Reveal Gastric Adenocarcinoma and Proximal Polyposis of the Stomach as a Familial Adenomatous Polyposis Variant, American Journal of Human Genetics. (2016) 98, no. 5, 830–842, 10.1016/j.ajhg.2016.03.001, 2-s2.0-84963558327, 27087319. 27087319 PMC4863475

[6] Saito-Diaz K. , Benchabane H. , Tiwari A. , Tian A. , Li B. , Thompson J. J. , Hyde A. S. , Sawyer L. M. , Jodoin J. N. , Santos E. , Lee L. A. , Coffey R. J. , Beauchamp R. D. , Williams C. S. , Kenworthy A. K. , Robbins D. J. , Ahmed Y. , and Lee E. , APC Inhibits Ligand-Independent Wnt Signaling by the Clathrin Endocytic Pathway, Developmental cell. (2018) 44, no. 5, 566–581.e8, 10.1016/j.devcel.2018.02.013, 2-s2.0-85042683098, 29533772. 29533772 PMC5884143

[7] El Hachem N. , Abadie C. , Longy M. , Colas C. , Fert-Ferrer S. , Leroux D. , Grandval P. , Prieur F. , Collonge-Rame M. , Faivre L. , and Fricker J. P. , Endoscopic Phenotype of Monoallelic Carriers of MUTYH Gene Mutations in the Family of Polyposis Patients: A Prospective Study, Diseases of the Colon & Rectum. (2019) 62, no. 4, 470–475, 10.1097/DCR.0000000000001323, 2-s2.0-85062607167, 30640315. 30640315

[8] Abraham S. C. , Nobukawa B. , Giardiello F. M. , Hamilton S. R. , and Wu T. T. , Fundic Gland Polyps in Familial Adenomatous Polyposis: Neoplasms With Frequent Somatic Adenomatous Polyposis Coli Gene Alterations, American Journal of Pathology. (2000) 157, no. 3, 747–754, 10.1016/S0002-9440(10)64588-9, 2-s2.0-0034495101, 10980114. 10980114 PMC1885693

[9] Abraham S. C. , Park S. J. , Mugartegui L. , Hamilton S. R. , and Wu T. T. , Sporadic Fundic Gland Polyps With Epithelial Dysplasia: Evidence for Preferential Targeting for Mutations in the Adenomatous Polyposis Coli Gene, American Journal of Pathology. (2002) 161, no. 5, 1735–1742, 10.1016/S0002-9440(10)64450-1, 2-s2.0-0036840842, 12414520. 12414520 PMC1850790

[10] Niu S. , Lucas E. , Molberg K. , Strickland A. , Wang Y. , Carrick K. , Rivera-Colon G. , Gwin K. , SoRelle J. A. , Castrillon D. H. , Zheng W. , and Chen H. , Morules but not Squamous Differentiation Are a Reliable Indicator of CTNNB1 (*β*-catenin) Mutations in Endometrial Carcinoma and Precancers, American Journal of Surgical Pathology. (2022) 46, no. 10, 1447–1455, 10.1097/PAS.0000000000001934, 35834400. 35834400

[11] Mitra A. and Matsukuma K. , Squamous Morules in a Fundic Gland Polyp: A Rare Benign Mimic of Well-Differentiated Neuroendocrine Tumor, American Journal of Clinical Pathology. (2022) 158, no. supplement_1, S71–S72, 10.1093/ajcp/aqac126.145.

[12] Sasaki A. , Yokoyama S. , Arita T. , Inomata M. , Kashima K. , and Nakayama I. , Morules With Biotin-Containing Optically Clear Nuclei in Colonic Tubular Adenoma, American Journal of Surgical Pathology. (1999) 23, no. 3, 336–341, 10.1097/00000478-199903000-00014, 2-s2.0-0033048875, 10078926. 10078926

[13] Boyraz B. , Sadow P. M. , Asa S. L. , Dias-Santagata D. , Nosé V. , and Mete O. , Cribriform-Morular Thyroid Carcinoma Is a Distinct Thyroid Malignancy of Uncertain Cytogenesis, Endocrine Pathology. (2021) 32, no. 3, 327–335, 10.1007/s12022-021-09683-0, 34019236. 34019236 PMC9353615

[14] Schlosnagle D. C. and Hardin R. D. , Squamous Morules in Gastric Mucosa, Journal of Clinical Gastroenterology. (1988) 10, no. 3, 332–334, 10.1097/00004836-198806000-00020, 2-s2.0-0023808915, 2980771. 2980771

[15] Jeon M. S. , Kim G. H. , Park D. Y. , Jeong J. H. , Kahng D. H. , Jang H. Y. , Choi J. H. , and Park E. K. , A Case of Squamous Metaplasia of the Stomach, Clinical Endoscopy. (2013) 46, no. 4, 407–409, 10.5946/ce.2013.46.4.407, 2-s2.0-84881035104, 23964341. 23964341 PMC3746149

[16] Yao S. and Deng X. , A Case Report of Gastric Squamous Cell Carcinoma Associated With Pancreatic Adenocarcinoma and Literature Review, Case Reports in Gastroenterology. (2025) 19, no. 1, 496–501, 10.1159/000546802, 40635759. 40635759 PMC12240571

